# 47149 Imaging Tools for Early Detection of Kidney Disease.

**DOI:** 10.1017/cts.2021.694

**Published:** 2021-03-30

**Authors:** Edwin J. Baldelomar, David Reichert, Kooresh Shoghi, Kevin M. Bennett

**Affiliations:** Washington University in St. Louis

## Abstract

ABSTRACT IMPACT: Chronic kidney disease (CKD) affects ˜15% of the US population and the majority of patients are diagnosed too late to benefit from early intervention. We are developing a new diagnostic imaging tool (RadioCF-PET) for the kidney to enable early detection of diseases and to monitor overall kidney health. OBJECTIVES/GOALS: Nephron mass, or the number of functioning nephrons, is a measure of the functional capacity of the kidney. RadioCF-PET may enable early detection of nephron loss in patients with or at risk of CKD before changes are clinically detectable, facilitating early interventions to improve outcomes in these patients. METHODS/STUDY POPULATION: RadioCF-PET, labeled with Cu-64, has the advantage of using sub pharmacological doses for imaging, carrying low risk and can be used with the FDA’s exploratory IND (eIND) mechanism for early in human testing. We are developing the technology to be used in pre-eIND toxicology and pharmacology studies. We are also developing other aspects of translational science to propel this technology toward translation, including: market analysis, critical path to market, customer discovery, and commercialization strategy. RESULTS/ANTICIPATED RESULTS: Milestone 1: Apply technology in mouse model study and in human kidneys rejected for transplant. Anticipated Result 1: PET signal from RadioCF-PET correlates with glomerular density in healthy and diseased model male mice (R2 = 0.98). RadioCF-PET signal correlates with glomerular number in a donated human kidney (R2= 0.78). Milestone 2: Application to federal funding (STTR) and gap funding mechanisms to enable pre-eIND studies. Anticipated Result 1: Application for funding will aid to clarify and validate our market analysis and commercialization strategy. Milestone 3: Continued research and development with the technology in new studies. Other Anticipated Results: Future work with RadioCF-PET will enhance technology performance in preparation for pre-eIND studies. DISCUSSION/SIGNIFICANCE OF FINDINGS: We foresee a large clinical and commercial potential for RadioCF-PET to provide precise, early monitoring in patients at risk for or with CKD. The two biggest hurdles for clinical translation are validating safety and proving efficacy. This work targets both issues to facilitate RadioCF-PET toward clinical translation.

